# Topological Approach to Void Finding Applied to the SDSS Galaxy Map

**DOI:** 10.3847/1538-4357/ad75fd

**Published:** 2024-10-28

**Authors:** Manu Aggarwal, Motonari Tonegawa, Stephen Appleby, Changbom Park, Vipul Periwal

**Affiliations:** 1 Laboratory of Biological Modeling/NIDDK, National Institutes of Health, Bethesda, MD, USA; manu.aggarwal@nih.gov, vipulp@niddk.nih.gov; 2 Asia Pacific Center for Theoretical Physics, Pohang, 37673, Republic of Korea; motonari.tonegawa@apctp.org, stephen.appleby@apctp.org; 3 Department of Physics, POSTECH, Pohang, 37673, Republic of Korea; 4 School of Physics, Korea Institute for Advanced Study, 85 Hoegiro, Dongdaemun-gu, Seoul, 02455, Republic of Korea; cbp@kias.re.kr

## Abstract

The structure of the low redshift Universe is dominated by a multiscale void distribution delineated by filaments and walls of galaxies. The characteristics of voids, such as morphology, average density profile, and correlation function, can be used as cosmological probes. However, their physical properties are difficult to infer due to shot noise and the general lack of tracer particles used to define them. In this work, we construct a robust, topology-based void-finding algorithm that utilizes Persistent Homology to detect persistent features in the data. We apply this approach to a volume-limited subsample of galaxies in the SDSS I/II Main Galaxy catalog with the *r*-band absolute magnitude brighter than *M*_*r*_ = −20.19, and a set of mock catalogs constructed using the Horizon Run 4 cosmological *N*-body simulation. We measure the size distribution of voids, their averaged radial profile, sphericity, and the centroid nearest neighbor separation, using conservative values for the threshold and persistence. We find 32 topologically robust voids in the SDSS data over the redshift range 0.02 ≤ *z* ≤ 0.116, with effective radii in the range 21−56 *h* ^−1^ Mpc. The median nearest neighbor void separation is found to be ∼57 *h* ^−1^ Mpc, and the median radial void profile is consistent with the expected shape from the mock data.

## 1. Introduction

The cosmic web, as traced by galaxies in the late Universe, is an interesting feature of the gravitational collapse of an initially Gaussian density field that naturally appears and evolves in the late-time universe (M. Jôeveer et al. 1978; S. A. Gregory & L. A. Thompson 1978; A. A. Klypin & S. F. Shandarin 1983; J. Gott et al. 1986; S. D. M. White et al. 1987; M. S. Vogeley et al. 1994b; J. R. Bond et al. 1996; J. M. Colberg et al. 2005; R. van de Weygaert & J. R. Bond 2008; N. I. Libeskind et al. 2018; S. Codis et al. 2018; C. Park et al. 2022). In three dimensions, matter collapses locally in an anisotropic manner, from underdensities onto two-dimensional sheets, then into one-dimensional filaments, and finally accreting into knots. The gravitational outfall from underdense regions generates large voids in the late universe, bounded by the two-dimensional walls generated as a result of the anisotropic collapse. Voids comprise the overwhelming majority of the spatial volume at *z* ≃ 0 (D. C. Pan et al. 2012).

The defining characteristic of a void is a dearth of matter, which makes it difficult to quantify and measure. We observe galaxies, which comprise a relatively sparse point distribution that is biased relative to the dark matter field. Regions of space that are not well sampled will be heavily affected by noise when attempting to infer properties of the underlying matter density field. In spite of these difficulties, voids have proved to be a valuable source of cosmological information, and they have been applied to the Alcock–Paczynski test (B. S. Ryden 1995; G. Lavaux & B. D. Wandelt 2012; P. M. Sutter et al. 2012, 2014), and other forms of cosmological parameter estimation (N. Hamaus et al. 2015, 2020; A. Kovács et al. 2022; S. Contarini et al. 2023, 2024; N. Schuster et al. 2023). Voids can also be used to test extensions to the standard model (E. Platen et al. 2008; J. Lee & D. Park 2009; E. G. P. Bos et al. 2012; D. Spolyar et al. 2013; E. Massara et al. 2015; Y.-C. Cai et al. 2015; I. Achitouv 2016; G. Pollina et al. 2019; G. Verza et al. 2019; K. C. Chan et al. 2019; S. Nadathur et al. 2020; A. Woodfinden et al. 2022).

To extract cosmological information from the distribution of voids, we must first measure their individual properties, such as volume and morphology. Numerous different void finders are employed within cosmology for this purpose (M. S. Vogeley et al. 1994a; H. El-Ad & T. Piran 1997; E. Platen et al. 2007; M. C. Neyrinck 2008; M. A. Aragon-Calvo et al. 2010; D. C. Pan et al. 2012; P. M. Sutter et al. 2015). They typically involve searching for underdense regions with a fixed shape template (spherical, ellipsoidal), or generating a set of polygons from galaxy positions, assigning a local density to these shapes according to their volume, and finally linking adjacent, low-density polygons to generate macroscopic structures (but see J. Shim et al. 2021b and J. Shim et al. 2023 for a void definition not assuming any geometry). They yield somewhat different morphologies, density profiles, etc., leading to some ambiguity in void properties. These different approaches are typically based on geometric assumptions and are fundamentally different from the mathematically rigorous topology computed by persistent homology (PH). The method of PH applies techniques developed in algebraic topology to find robust lacunae in noisy, discrete data sets. PH makes no assumptions about the geometry of voids a priori, which is important since the real Universe consists of regions of relatively low density that are more often polyhedral than spherical (V. Icke & R. van de Weygaert 1987; M. C. Neyrinck 2008). Indeed, we do not expect voids to be spherical because the critical points of the Gaussian initial density field are generically ellipsoidal (J. M. Bardeen et al. 1986). As underdense regions grow in volume due to gravitational collapse onto surrounding walls, they have a tendency to become increasingly spherical. However, at late times they merge to form complex morphological structures (R. K. Sheth & R. van de Weygaert 2004; E. Jennings et al. 2013).

In this work, we apply the PH methodology to the SDSS main galaxy sample, inferring a number of key properties of voids in the low redshift Universe: their size distribution, averaged radial profiles, sphericity, and the pairwise distribution of void voxels. We perform a comparative study between the data and mock galaxy catalogs and between our approach and another void finder in the literature.

The paper proceeds as follows. In Section 2 we provide a brief and nontechnical review of some of the important aspects of the topological methodology employed in this work. In Section 3 we introduce the galaxy catalog from which voids are extracted and the mock data that is used for comparative purposes. Section 4 contains the main results of our analysis; the properties of voids found using PH in the data and mock catalogs. We compare our results with other void finders in the literature in Section 4.2, and discuss our findings in Section 5. The Appendix contains some of the more technical details of the numerical algorithms used in the main body of the paper.

## 2. Introduction to Persistent Homology

We begin with a brief review of some of the important underlying ideas used in topological data analysis, in particular for the void-finding algorithm applied to galaxy data in Section 4. The discussion is intended to be nontechnical; further details can be found in the appendices.

**Figure 1.**
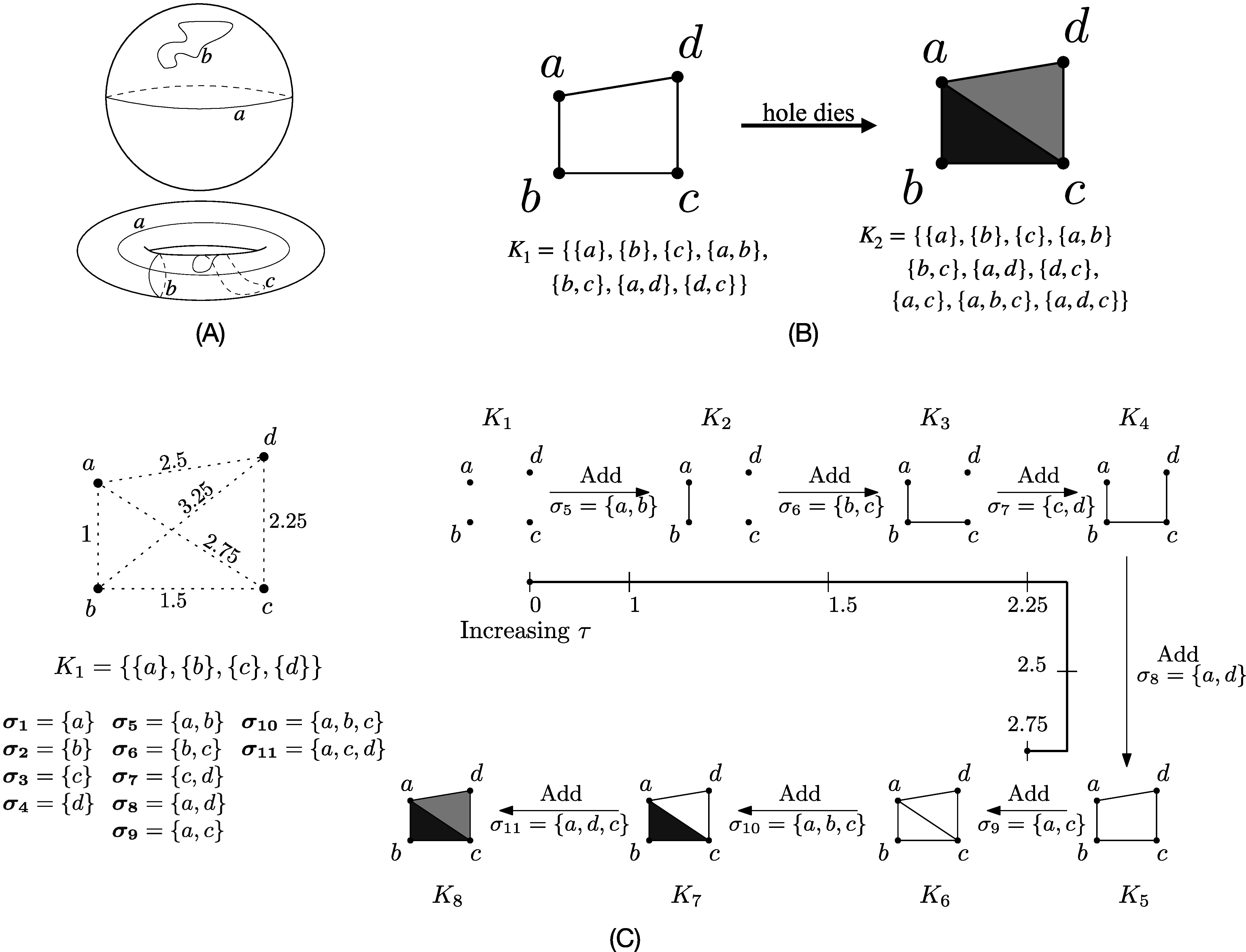
(A) Top panel shows two cycles *a* and *b* on the surface of a sphere. Both can be continuously deformed along the surface of the sphere to the same point. Any cycle on the surface of a sphere can contract to a point. The bottom panel shows three cycles on the surface of a 2-torus. Cycle *c* contracts to a point. Cycles *a* and *b* are noncontractible and cannot be deformed to each other. Any noncontractible cycle on the surface of the torus can deform to either *a* or *b*. (B) A hole dies when its triangulation gets filled in. The hole in simplicial complex *K*_1_ dies when edge {*a*, *c*} and triangles {*a*, *b*, *c*} and {*a*, *d*, *c*} are added to it to give a new complex *K*_2_. (C) (from M. Aggarwal et al. 2023) Vietoris–Rips filtration on a discrete set of points. The simplicial complex at a given value of *τ* is a collection of all simplices with diameter at most *τ*. Number of holes is initially 0. One hole is born at a spatial scale of *τ* = 2.5 when edge {*a*, *d*} is added. Another is born when {*a*, *c*} is added at *τ* = 2.75. Both holes get filled in, or die, at *τ* = 2.75 when triangles (2-simplices) are added to the simplicial complex. The first hole has persistence 2.75 − 2.5 = 0.25. The second hole dies at the same spatial scale it is born on, and so has persistence 0.

A homology group is a collection of sets of cycles such that any two cycles in the same set can be continuously deformed into one another, and those in different sets cannot. For example, all cycles on the surface of a sphere can be shrunk or contracted continuously along its surface to a point (see Figure 1(A) top panel). On the other hand, Figure 1 also shows three homologically distinct cycles on the surface of a 2-torus (Figure 1(A) bottom panel). Cycle *c* can contract to a point, whereas cycles *a* and *b* can neither contract to a point nor can be deformed to each other. We say that cycle *c* is contractible (belonging to the trivial homology), and *a* and *b* are noncontractible cycles in different equivalence classes of the homology group of dimension one (H_1_). Moreover, any noncontractible cycle on the surface of a torus can be deformed either to *a* or to *b*. Hence, there are exactly two topologically distinct holes in this shape, and *a* and *b* are examples of representative boundaries for these. This gives a classification of the shape of the surface of a 2-torus based on the number of homologically distinct noncontractible cycles on its surface. In this example, we discussed cycles on the surface of the torus, which are also called cycles of dimension one and belong to the homology group of dimension one. Similarly, the homology group of dimension two (H_2_) is the collection of sets of noncontractible cycles of dimension two. Intuitively, they can be thought of as noncontractible surfaces around voids in a point cloud (i.e., a discrete set of points) embedded in a three-dimensional Euclidean space.

In real-life applications, experimental data are often discrete observations that can be embedded as a point cloud and not a smooth manifold. Homology groups of a point cloud can be computed by constructing simplices. Briefly, a *n*-simplex is a set of *n* + 1 points. For example, 0-simplices are points, 1-simplices are edges, 2-simplices are triangles, and 3-simplices are tetrahedrons. A collection of simplices is called a simplicial complex. Figure 1(B) shows two simplicial complexes. Complex *K*_1_ has a hole of dimension 1 and the edges in the simplex form a representative boundary around this hole. The complex *K*_2_ contains triangles {*a*, *b*, *c*} and {*a*, *c*, *d*} along with the simplices of *K*_1_. Visually, these are shown as the two filled-in triangles in *K*_2_ in the figure. Naively, we can note that the hole in *K*_1_ is filled in when its triangulation is added to the complex. We say that the hole in *K*_1_ dies when the triangles {*a*, *b*, *c*} and {*a*, *c*, *d*} are added to it. Simplicial homology rigorously defines this notion of holes and their birth and death using algebraic topology, and it generalizes to high dimensions. Readers interested in the mathematical and computational details are directed to H. Edelsbrunner & J. L. Harer (2022).

When a discrete set is embedded in a Euclidean space, we can compute its homology groups at different spatial scales. Vietoris–Rips filtration (VR-filtration) is a commonly used construction of simplicial complexes at different spatial scales. The simplicial complex at spatial scale *τ* is defined as the collection of all simplices that have *τ* as the maximal pairwise distance between their points. As the spatial scale changes, the simplicial complex changes, and there might be birth and death of holes (Figure 1(C)). PH computes these births and deaths, and they are plotted as persistence diagrams (PDs).

They give a global multiscale overview of the topology of the shape of the discrete data set. The persistence of a topological feature, or a hole, is defined as the duration *τ* between its death and birth. Features with a large persistence are usually deemed as topologically significant. Due to its generality and computation of robust topological features in noisy data sets across multiple scales, PH has found useful applications in areas as diverse as neuroscience (P. Bendich et al. 2016), computational biology (M. R. McGuirl et al. 2020), natural language processing (X. Zhu 2013), the spread of contagions (D. Taylor et al. 2015), cancer diagnosis (M. Nicolau et al. 2011; P. Lawson et al. 2019), material science (M. Kramár et al. 2013), computer graphics (R. Brüel-Gabrielsson et al. 2020), cosmology (A. L. Melott 1990; K. R. Mecke et al. 1994; M. Kerscher et al. 1998; T. Sousbie et al. 2011; C. Park et al. 2013; J. Feldbrugge et al. 2019; P. Pranav et al. 2019; G. Wilding et al. 2021; P. Pranav 2021; R. van de Weygaert et al. 2011) among many others. In the context of cosmology, homology groups are associated with different cosmic environment types as follows. Connected components (zero-dimensional homology groups, H_0_), loops (one-dimensional homology groups, H_1_), and low-density three-dimensional volumes (two-dimensional homology groups, H_2_) are analogous to galaxy clusters, closed loops of filaments, and cosmic voids, respectively(X. Xu et al. 2019). Locations of voids can be estimated by computing representative boundaries of topologically significant H_2_ features. However, the representatives are not unique by definition (G. Carlsson 2009). We define tight representatives as those with shorter lengths (fewer number of simplices in the boundary). Computing optimal tight representatives is computationally intractable, even for a few thousand points. M. Aggarwal et al. (2023) developed an algorithm for large data sets that computes representatives that may not be optimal but are significantly shortened as compared to those obtained from the matrix reduction algorithm by default. Using it, we find geometrically precise boundaries around significant topological features in the SDSS and HR4 data sets, which have more than a hundred thousand galaxies.

## 3. Data

### 3.1. SDSS Spectroscopic Sample

To construct void catalogs, we require a galaxy catalog, preferably with certain desirable properties. The data should be contiguous on the sky, have high angular completeness, and be volume limited. Three-dimensional void construction may be severely impaired by the presence of a significantly varying angular selection function on the sky, and similarly by systematic variation in the number density with redshift. We also require a point distribution that occupies a large volume to generate a statistical sample that can be useful for cosmology and also to capture the morphology of the largest voids. Given these requirements, the SDSS I+II DR7 Main Galaxy catalog is ideally suited for our purpose. Specifically, we adopt the Korea Institute for Advanced Study Value Added Galaxy Catalog (KIAS-VAGC; Y.-Y. Choi et al. 2010), which is based on the original catalog (M. R. Blanton et al. 2005) but is supplemented with additional redshift information from the Zwicky catalog (E. E. Falco et al. 1999), the IRAS Point Source Catalog Redshift Survey (W. Saunders et al. 2000), the Third Reference Catalog of Bright Galaxies (G. de Vaucouleurs et al. 1991) and the Two-Degree Field Galaxy Redshift Survey (M. Colless et al. 2001), together with the updated angular selection function.

**Figure 2.**
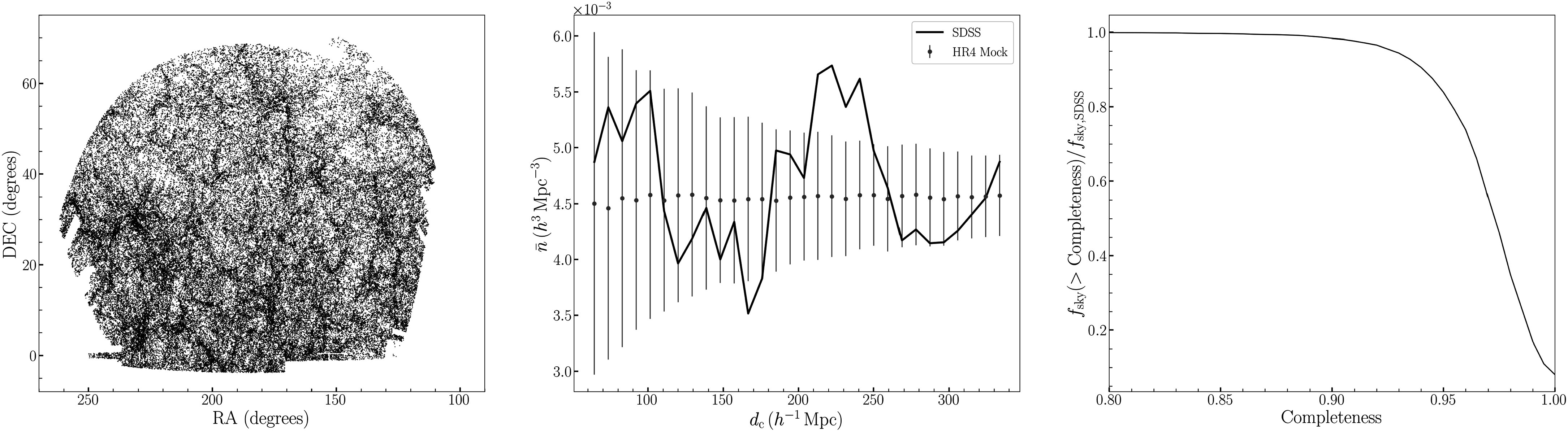
The SDSS galaxy point distribution as a function of angular coordinates on the sky (left panel). The number density $\bar{n}$ of the SDSS volume-limited sample as a function of comoving distance is presented in the middle panel (solid black line), along with the mean and rms values of the mocks (blue points/error bars). In the right panel, we present the fraction of the SDSS footprint as a function of its angular completeness. approximately 90% of the footprint has completeness greater than 0.95.

The catalog contains 593,514 redshifts, with *r*-band Petrosian magnitudes in the range 10 < *r*_p_ < 17.6. Since we require contiguous data, we remove the three southern stripes and the Hubble Deep Field region. Approximately 90% of the angular mask has completeness higher than 95%. In Figure 2 (right panel), we present the completeness of the angular selection function as a fraction of the area occupied on the sky. We also present the full galaxy catalog in angular coordinates (R.A. versus decl., left panel). The redshift and corresponding *r*-band absolute magnitude cuts are 0.02 ≤ *z* ≤ 0.116 and *M*_*r*_ ≤ −20.19 respectively^6^ 
^6^
The term $+5\mathrm{log}h$ is dropped, i.e., *h* = 1 is used, in the conversion from apparent magnitudes to absolute magnitudes. for the volume-limited subsample that we select for our analysis. The evolution correction for the absolute magnitudes, *E*(*z*) = 1.6(*z* − 1), is applied (M. Tegmark et al. 2004). In the middle panel of Figure 2, we present the number density of this volume-limited sample as a function of comoving distance, assuming a flat ΛCDM cosmology with Ω_*m*_ = 0.3. The black solid line is the number density of the SDSS data, and the blue points/error bars are the mean and rms of the mocks, described in Section 3.2.

For the purposes of our study, the SDSS galaxies are not weighted by the angular selection function at any point during the PH calculation and subsequent void-finding algorithm. Because the completeness of the value-added catalog is so high in this particular data set, we do not expect the angular selection function to have any significant effect on the resulting properties of the voids. This may be an issue for higher redshift data, where the completeness on the sky is generally lower, and data quality is worse.

### 3.2. Mock Galaxy Data

We compare the void catalog from the SDSS data to mock catalogs designed to match the clustering properties of the observed galaxy distribution. For this purpose, we adopt the Horizon Run 4 (HR4) simulation. HR4 is a cosmological scale *N*-body simulation in which 6300^3^ cold dark matter particles were gravitationally evolved in a $V={\left(3150\,{h}^{-1}\,\mathrm{Mpc}\right)}^{3}$ box using a modified version of GOTPM code.^7^ 
^7^
The original GOTPM code is introduced in J. Dubinski et al. (2004). A review of the modifications introduced in the Horizon Run project can be found at https://astro.kias.re.kr/~kjhan/GOTPM/index.html.
 The WMAP5 cosmology (E. Komatsu et al. 2009) is used for this simulation. Details of the simulation can be found in J. Kim et al. (2015). Dark matter halos and subsequently mock galaxy catalogs are constructed in S. E. Hong et al. (2016) using a most-bound halo particle-galaxy correspondence algorithm, with satellite galaxy survival time after mergers calculated using a modified model of C. Y. Jiang et al. (2008)

\begin{eqnarray*}\displaystyle \frac{{t}_{\mathrm{merge}}}{{t}_{\mathrm{dyn}}}=\displaystyle \frac{\left(0.94{\epsilon }^{0.6}+0.6\right)/0.86}{\mathrm{ln}\left[1+({M}_{\mathrm{host}}/{M}_{\mathrm{sat}})\right]}{\left(\displaystyle \frac{{M}_{\mathrm{host}}}{{M}_{\mathrm{sat}}}\right)}^{1.5},\end{eqnarray*}

where *t*_dyn_ is a dynamical timescale—the orbital period of a virialized object—*M*_host_, *M*_sat_ are the host halo and satellite masses, and *ϵ* is the circularity of the satellite’s orbit at the time of merger. The particle and halo mass resolutions of the simulation are 9.0 × 10^9^ *h* ^−1^ *M*_⊙_ and 2.7 × 10^11^ *h* ^−1^ *M*_⊙_, respectively.

We use the *z* = 0 snapshot box to generate mock catalogs—*N*_r_ = 360 observers are placed in the box, maximally separated to ensure the mock data does not overlap. To reproduce the survey geometry of the SDSS data we use, the SDSS angular footprint is applied relative to each observer placed at the corners. A global mass cut is applied to all galaxies in the snapshot box to ensure that the average number density $\bar{n}$ matches that of the SDSS data, $\bar{n}=4.6\times {10}^{-3}\,({h}^{3}\,{\mathrm{Mpc}}^{-3})$. For absolute magnitude-limited catalogs with a high magnitude selection function like the one used in this work, simulated data with a mass cut typically provides a very good match to a magnitude cut.

The galaxy positions that we obtain from spectroscopic redshifts are shifted by the Doppler effect due to the radial component of their own motions. This effect is called the redshift space distortions (N. Kaiser 1987), and the modulation from the real-space position ***r*** to the redshift-space (observed) position ***s*** caused by the galaxy velocity ***v*** is expressed as

\begin{eqnarray*}{\boldsymbol{s}}={\boldsymbol{r}}+\displaystyle \frac{1}{{aH}}{\hat{{\boldsymbol{e}}}}_{\parallel }({\boldsymbol{v}}\cdot {\hat{{\boldsymbol{e}}}}_{\parallel }),\end{eqnarray*}

where *a*, *H*, and ${\hat{{\boldsymbol{e}}}}_{\parallel }$ are the scale factor, Hubble parameter, and the unit vector along the joining line between a galaxy and the observer. We apply this correction to each simulated galaxy to generate the redshift-space mock catalogs that are consistent with the observation data. We only perform the PH analysis for the redshift-space mocks and do not repeat it for the real space as the purpose of mocks is to check the consistency between observational data and the predictions from the ΛCDM cosmology simulation. Studying the difference in void statistics for these two spaces is beyond the scope of the present study and will be pursued in future works.

## 4. Results

### 4.1. Topologically Significant Voids Computed for SDSS DR7 and Mocks Exhibit Similar Properties

**Figure 3.**
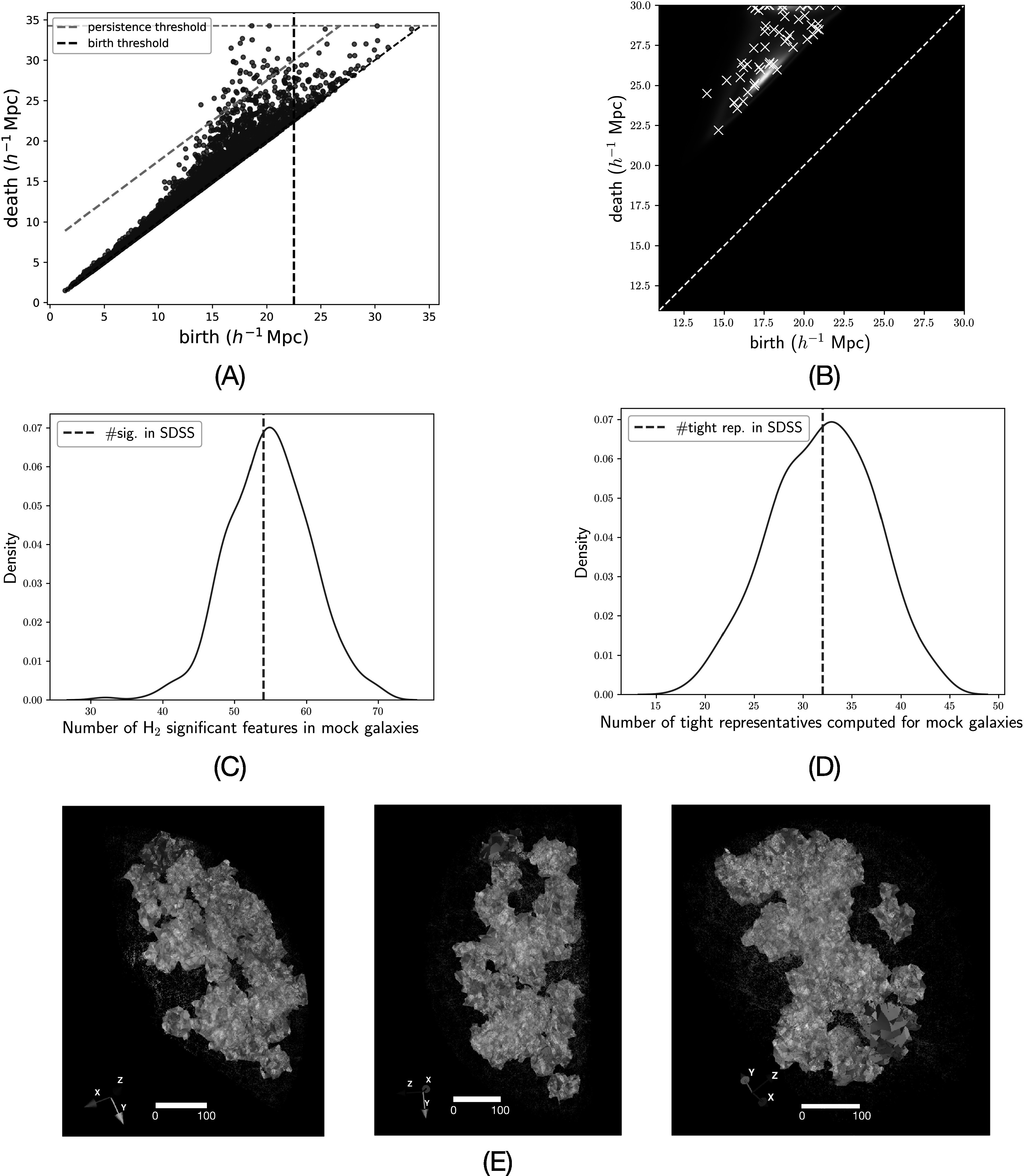
Topology of the mocks agrees with SDSS. (A) H_2_ PD for SDSS DR7 computed until a threshold of 30 *h* ^−1^ Mpc. Significant features are defined as those above the orange dashed line (persistence threshold of 7.5 *h* ^−1^ Mpc) and to the left of the black dashed line (birth threshold of 22.5 *h* ^−1^ Mpc). (B) kernel density estimation plot of significant features over all mocks. Significant features in SDSS are shown by white x markers. (C) The number of significant H_2_ features. (D) Distribution of numbers of computed tight H_2_ representatives in mocks. (E) Three views of computed tight H_2_ representatives in SDSS. One of the voids is highlighted in blue color.

We compute the PH of SDSS DR7 for Vietoris–Rips filtration (VR-filtration, see Appendix A) up to a threshold for the spatial scale of 36 *h* ^−1^ Mpc. Figure 3(A) shows the resulting H_2_ PD, and in particular presents an important characteristic of the cosmic web—it is a multiscale phenomenon. Voids are born, die, and exhibit persistence thresholds over the entire range of scales that can be reasonably probed by the SDSS data, from 1 *h* ^−1^ Mpc to 50 *h* ^−1^ Mpc. We define a feature to be significant if it has persistence at least 7.5 *h* ^−1^ Mpc (persistence threshold) and is born before 22.5 *h* ^−1^ Mpc (birth threshold). We explain these choices in detail in Appendix D. Subsequently, there are 57 significant topological features in SDSS. Based on our choice of thresholds, it suffices to compute PH up to 30 *h* ^−1^ Mpc to determine the significant features in all of the mocks. This reduces computational run time by more than a factor of three as compared to the computation up to 36 *h* ^−1^ Mpc. Computation costs for computing PH up to 30 *h* ^−1^ Mpc and representative boundaries of nontrivial features are shown in Appendix G.1. With our choice, we are selecting objects that are present at scales $\gtrsim { \mathcal O }(10\,{h}^{-1}\mathrm{Mpc})$, which are those typically parsed by the cosmology community.

Figure 3(B) shows a kernel density estimation (KDE) plot of distributions of births and deaths of significant features in all mocks, along with those in SDSS (white x markers). The overlay shows that H_2_ topology is similar across mocks and SDSS. Figure 3(C) shows that the number of significant features in SDSS agrees with the median of the number of significant features in mocks. A total of 32 tight representatives around single significant voids were computed for SDSS. The number of tight representative boundaries is less than that of significant features due to generally larger birth scales and unchanged death scales after the shortening procedure. We explain this in Appendix C. Figure 3(D) shows the distribution of the number of tight representatives around single voids computed for mocks. Figure 3(E) shows three different views of all the representative boundaries computed in SDSS. We visually observe that the computed boundaries are polyhedral and not necessarily convex.

**Figure 4.**
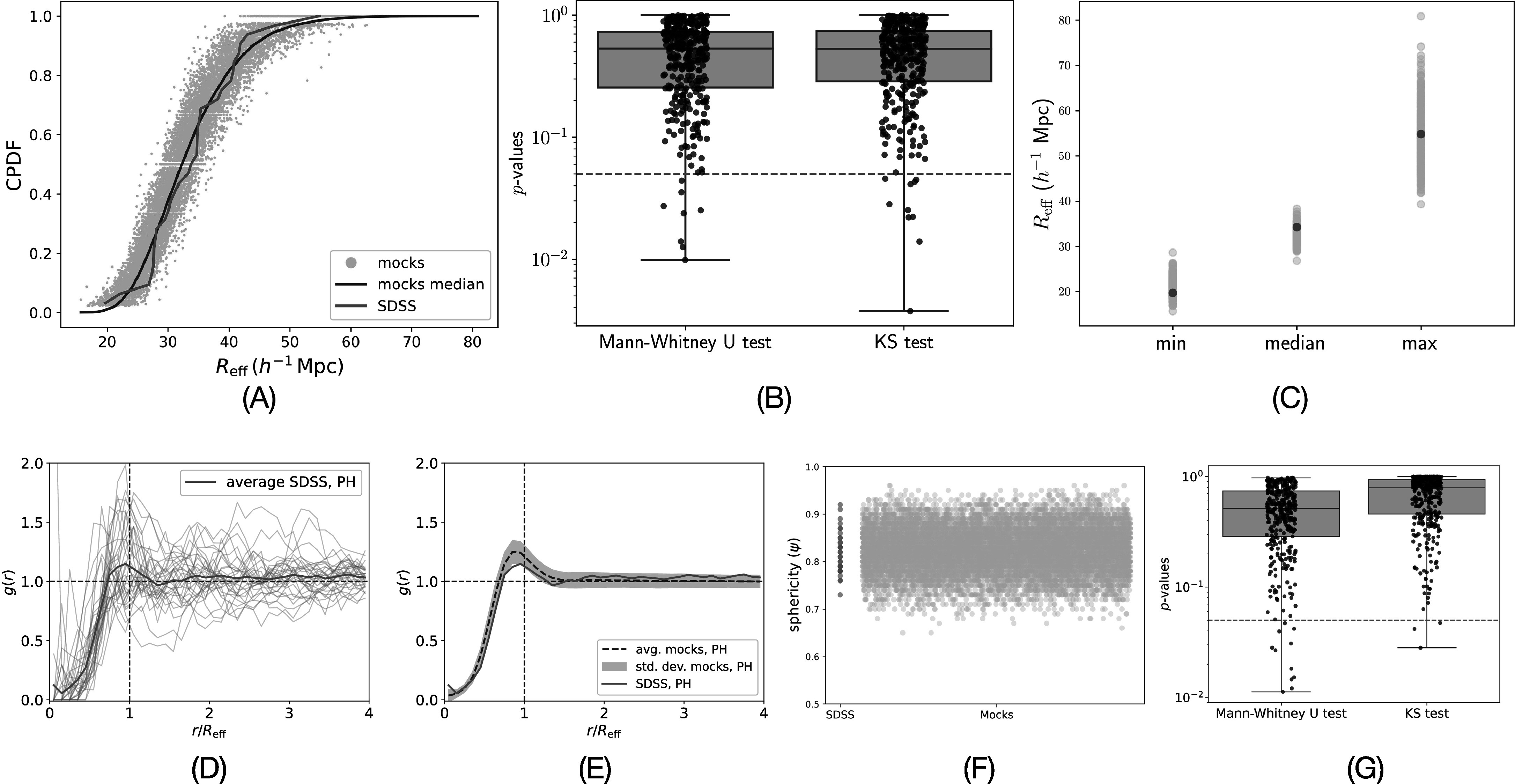
SDSS and the 360 mocks exhibit similar morphology. (A) Cumulative probability density function (CPDF) of *R*_eff_ for SDSS (red) and all mocks (turquoise). (B) *p*-values from Mann–Whitney U and KS significance tests comparing the distribution of *R*_eff_ of voids in SDSS to the distributions of *R*_eff_ of voids in the mocks. The majority are greater than 0.05 (red dashed line). (C) Minimums, medians, and maximums of distributions of effective radii of voids computed for mocks (turquoise) and SDSS (red). (D) Radial distribution functions of each void that is computed for SDSS. The average of radial distributions over all voids is shown in a bold red line plot. (E) Averages of radial distribution functions of voids computed for mocks follow a similar pattern to the average of the voids in SDSS. (F) *p*-values from Mann–Whitney U and KS significance tests show that the sphericities of voids is less than 1 but greater than 0.6. (G) *p*-values from comparing distributions of sphericities of voids in mocks to that in SDSS.

We compute the effective radius (*R*_eff_) of each void as the radius of the sphere with the same volume as the convex hull of its computed representative boundary. Cumulative probability density functions of *R*_eff_ distribution of all voids in SDSS are compared to that of mocks in Figure 4(A). The distribution of *R*_eff_ of the voids computed for SDSS is compared to that of each of the 360 mocks using the Mann–Whitney U test and Kolmogorov–Smirnov (KS) test. Figure 4(B) shows that the distribution of *R*_eff_ of the voids in the majority of the mocks is not significantly different from that of SDSS (*p*-value ≫ 0.05).

Figure 4(C) shows that the minimums of the effective radii computed for the mocks range from approximately 15 to 30 *h* ^−1^ Mpc, and the medians range from 30 to 40 *h* ^−1^ Mpc, matching closely to SDSS. The maximums for the mocks, however, show a wider range of approximately 40–80 *h* ^−1^ Mpc.

Since computed H_2_ representatives are polyhedral, that might be very different from spherical approximations of boundaries of voids. For additional validation we compute the radial distribution function *g*(*r*) for each void with the centroid of its computed representative boundary as the reference point. Briefly, *g*(*r*) is the ratio of the density of shells of thickness 2 *h* ^−1^ Mpc around the centroid of the void to the density of shells containing a random point distribution (≈10^6^ points) distributed uniformly in the SDSS footprint. It is normalized by the mean value of the mocks at *r* = 4*R*_eff_, and the radii of the shells are finally normalized by *R*_eff_. Hence, *g*(*r*) > 1(*g*(*r*) < 1) indicates overdense(underdense) regions. This ratio accounts for the fact that at large distances from the centroid location, the shells will hit the boundary of the survey. So, the density of the galaxies will artificially drop but so will the density of random points.

Figure 4(D) shows the *g*(*r*) profiles of each void in SDSS for which a representative boundary was computed using PH. On average, *g*(*r*) rises for *r* < *R*_eff_ with a peak very close to *r* = *R*_eff_. Figure 4(E) shows that the averages of *g*(*r*) profiles of all mocks present a similar profile to the average of the SDSS. We note that the peak is not exactly at *r* = *R*_eff_ presumably because the voids in the Universe are not exactly spherical. We quantify this by computing sphericity (*ψ*) as the ratio of $4\pi {R}_{\mathrm{eff}}^{2}$ to the surface area of the computed polyhedral boundary. Figure 4(F) shows that the sphericity of all voids is less than 1 but more than 0.6. The average sphericity of the voids is around 0.8 in every configuration (SDSS and the 360 mocks). The distribution of the sphericities of the voids in the 360 mocks is compared to the distribution of those in SDSS using the Mann–Whitney U test and the Kolmogorov–Smirnov (KS) test. Figure 4(G) shows that the majority of mocks do not show significant differences (*p*-value > 0.05) in the sphericities of the voids as compared to SDSS. We expect voids to be aspherical because of the complex morphological structure of the cosmic web, and also due to intrinsic anisotropic observational artifacts such as redshift space distortion and the Alcock–Paczynski effect.

**Figure 5.**
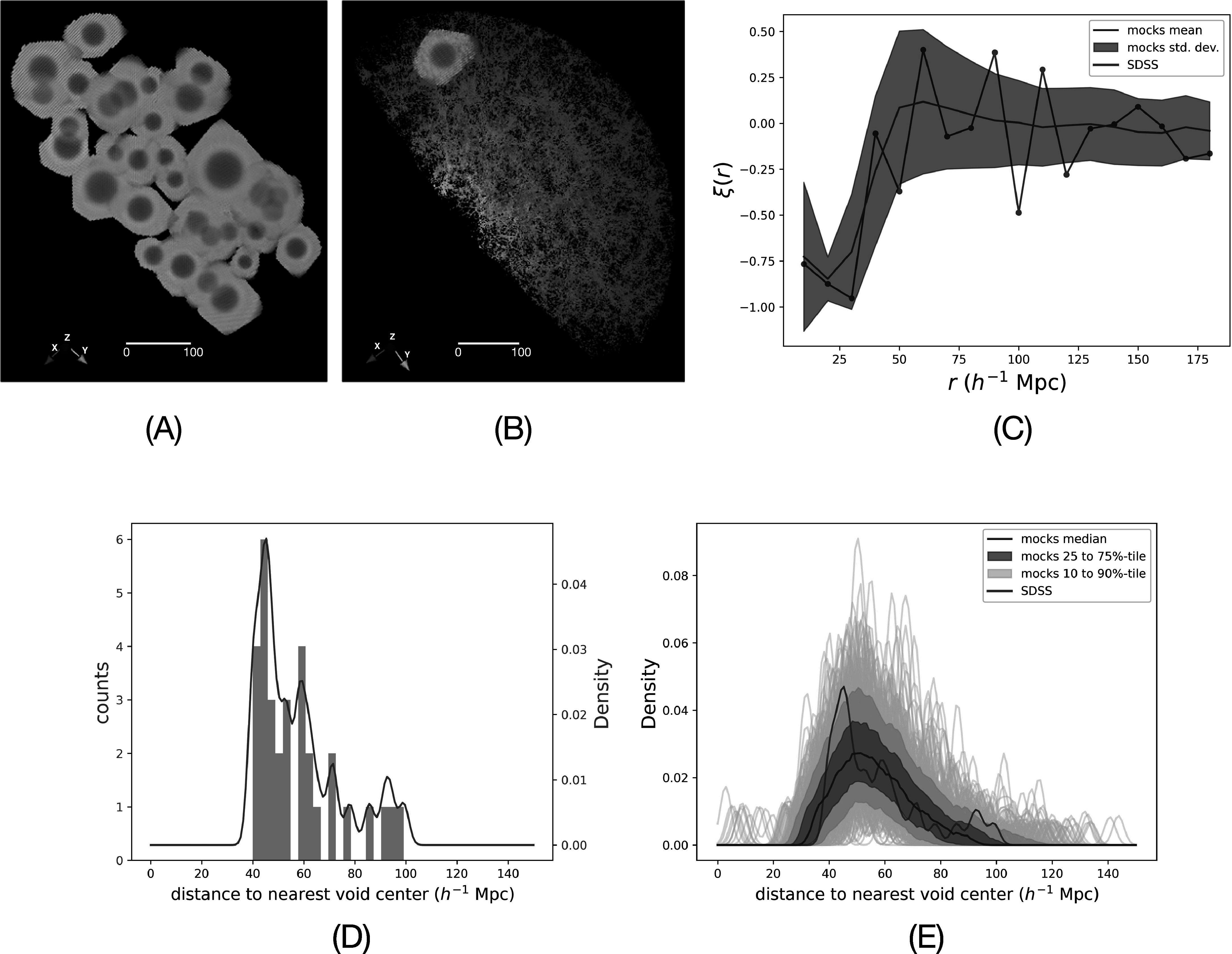
Characteristic distances between nearest void centers. (A) All cubic voxels that are inside voids computed for SDSS. (B) An example of cubic voxels inside one of the computed voids in SDSS. The color scheme is scaled based on the distance of voxels from the centroid of the void it is in. (C) The two-point correlation function of the void central positions. The red line represents the SDSS data, while the blue line and area indicate the mean and standard deviation from mocks. (D) Distribution of distances of the nearest void center to all void centers. The KDE of the distribution is shown in red. (E) KDE of the distributions of distances of the nearest void center to all void centers for the mocks (turquoise). The median of the KDEs for mocks is shown in blue.

Figures 5(A) and (B) show the PH voids computed in SDSS. For each void center, we compute the distance of the nearest void center from it. Figure 5(C) shows the two-point correlation function of void centers. We use the Landy–Szalay estimator (S. D. Landy & A. S. Szalay 1993), $\xi (r)\,=\tfrac{{DD}-2{DR}+{RR}}{{RR}}$, where *DD* takes the pair count of void centers in separation bins [*r* − Δ*r*, *r* + Δ*r*], *RR* is the pair count of random points distributed within the SDSS survey volume, and *DR* is the cross pair count between void centers and randoms. The number of random points is set to be sufficiently large, and *DR* and *RR* are normalized to *DD* accordingly. We observe that *ξ*(*r*) is peaked at ∼50–60 *h* ^−1^ Mpc and approaches zero as separation *r* increases. This means that the separation of voids is typically ∼60*h* ^−1^ Mpc, which is also observed in Figure 5(D). The drop below 50 *h* ^−1^ Mpc is due to the size of voids, i.e., the exclusion of other voids (N. Hamaus et al. 2014; T. Baldauf et al. 2016; J. Shim et al. 2021a). Figure 5(D) shows the distribution of nearest neighbor distances for the SDSS. The KDE of this distribution is shown in red; the majority of nearest void centers are within 40–60 *h* ^−1^ Mpc. Figure 5(E) shows KDEs of similar distributions for all of the mocks (turquoise). Although noisy due to a low number of objects in each realization, we find that the peak in the median of the KDEs of mocks (blue) matches with the peaks in the KDE of the SDSS (red).

The measured two-point statistics of the void centers—nearest neighbor separation and correlation function—present behavior that is typical of critical points (S. L. Lumsden et al. 1989). The anticlustering (“exclusion”) regime of the correlation function *r* < 50 *h* ^−1^ Mpc is related to our choice of birth and persistence thresholds because these indirectly determine the comoving size of the voids. In terms of critical points of some underlying continuous field traced by the galaxies, the exclusion zone can be understood as the typical length scale required for the curvature of the field to change signs twice between adjacent voids (J. Shim et al. 2021a).

### 4.2. Comparison with Other SDSS Void Catalogs

K. A. Douglass et al. (2023) compute and compare void catalogs for SDSS DR7 using two popular classes of void-finding algorithms, VoidFinder (H. El-Ad & T. Piran 1997) and *V* ^2^ (M. C. Neyrinck 2008). The former computes voids by first expanding spheres centered at locally empty regions until they are bounded by a threshold number of galaxies, and these spheres are then merged to define voids (K. A. Douglass et al. 2023). The latter first computes a three-dimensional Voronoi tessellation of the distribution of galaxies, then combines computed Voronoi cells into zones using watershed segmentation, which are finally merged into voids (K. A. Douglass et al. 2023). Since VoidFinder does not compute voids with an effective radius larger than 30 *h* ^−1^ Mpc (K. A. Douglass et al. 2023) and PH computes many larger voids, we only compute *V* ^2^ voids in the adopted KIAS-VAGC in this study for comparison with the computed PH voids.

**Figure 6.**
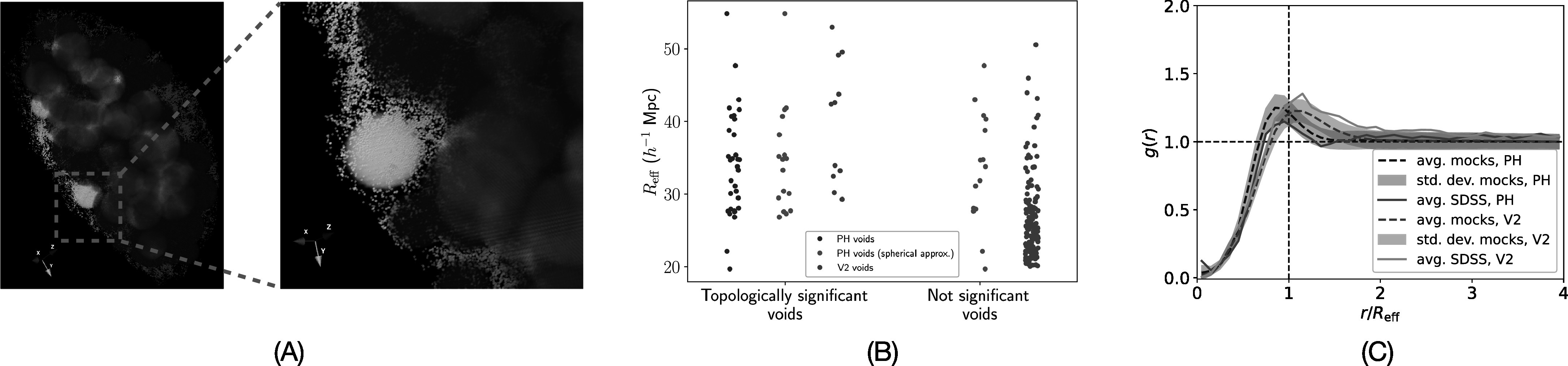
Comparing PH and *V* ^2^ voids. (A) PH voids in white and *V* ^2^ voids in blue. PH finds a void not found by *V* ^2^. (B) Distributions of *R*_eff_ of *V* ^2^ and PH voids, categorized on the basis of whether their representative boundaries contain topologically significant features. The majority of *V* ^2^ voids do not contain topologically significant features. (C) *g*(*r*) of PH and *V* ^2^ for SDSS and mocks.

We compute *V* ^2^ voids using the VAST toolbox (K. A. Douglass et al. 2022) with settings *H*_0_ = 100 *h* km s^−1^ Mpc^−1^ and Ω_*m*_ = 0.3. A total of 419 voids are reported with an effective radius at least 10 *h* ^−1^ Mpc. We consider voids with *R*_eff_ at least 20 *h* ^−1^ Mpc, resulting in 150 voids. Figure 6(A) shows spherical approximations of PH voids and *V* ^2^ voids based on *R*_eff_. The zoomed-in panel on the right in the figure shows a PH void that is not found by *V* ^2^.

We first compare the number of topologically significant voids. We say that a PH void is topologically significant if the smallest hyperrectangle around its representative boundary contains at least one significant feature. For a *V* ^2^ void, we compute the number of significant features in the smallest hyperrectangle around the sphere of the void’s effective radius centered at its centroid. For a fair comparison, we do the same for every PH void, and these are labeled as “spherical approximation.” Figure 6(B) shows that only 11 out of the 150 *V* ^2^ voids (green points) are topologically significant. On the other hand, all of the PH voids are topologically significant (blue points). Only around half of the spherical approximations of PH voids do not contain a significant feature. However, they are still more than the number of topologically significant *V* ^2^ voids. We further note that the discrepancy between the number of significant PH voids and significant spherical approximations of PH voids indicates that some of the PH voids are aspherical. Finally, we compare *g*(*r*) profiles for PH and *V* ^2^ voids in the SDSS and mocks in Figure 6(C). We note that the peak of *g*(*r*) for PH voids occurs at *r* < *R*_eff_ (at $\tfrac{r}{{R}_{\mathrm{eff}}}=0.95$) whereas for *V* ^2^ voids it occurs at *r* > *R*_eff_ (at $\tfrac{r}{{R}_{\mathrm{eff}}}=1.15$). This indicates that voids inferred using the two different methodologies possess different morphologies.

## 5. Discussion

We have introduced a void-finding algorithm that is based on the rigorous mathematics of PH and applied it to the SDSS DR7 main galaxy sample and a set of mock catalogs constructed from a cosmological scale dark matter simulation. Certain user inputs are required to define voids, and in this work, a birth threshold of *τ*_*u*_ = 22.5 *h* ^−1^ Mpc and persistence level *p* = 7.5 *h* ^−1^ Mpc are used to select topologically robust objects from the galaxy point distribution. We find representative boundaries around 32 unique voids that satisfy the criteria imposed, comprising a total volume fraction of 0.26 of the SDSS footprint over the redshift range 0.02 ≤ *z* ≤ 0.116. The physical properties of the voids have been ascertained, chiefly their radial profiles, effective radii distributions, the nearest neighbor separation, two-point correlation function, and sphericity. We find a range of sizes between 21−56 *h* ^−1^ Mpc, and a median nearest neighbor separation of ∼57 *h* ^−1^ Mpc. The properties of these objects will depend on the choice of birth threshold and persistence. The SDSS voids show excellent agreement with the same quantities extracted from the mock catalogs, indicating that the large-scale distribution of matter in the observed Universe closely matches our expectations from simple cold dark matter gravitational physics. This is a nontrivial result—the mock galaxies have been selected to match the two-point statistics of the SDSS, but the spatial distribution and morphological properties of voids carry information beyond the power spectrum. The void profiles *g*(*r*), obtained using PH and the *V* ^2^ algorithm, are in reasonable agreement, indicating that the void profile is a robust statistical quantity that can be used for cosmological parameter estimation. However, the peak of *g*(*r*) occurs at mildly different *r* values for the two algorithms, indicating that the nonspherical nature of the voids will play some role in determining the average shape.

The topological objects defined in this work are constructed from the observed point distribution rather than a smoothed density field inferred from the galaxy positions. This makes it difficult to analytically relate the void properties to cosmological parameters, since the standard cosmological model is predicated on a fluid description of matter. However, there are a number of interesting avenues that remain to be explored. First, the sensitivity of the void properties to galaxy bias and cosmological parameters can be tested by applying our algorithm to other mock galaxy and dark matter data. We expect only mild dependence on galaxy bias, since all galaxies should trace the same wall-like structures on large scales. The cosmological parameters Ω_m_ *h* ^2^ and *n*_s_ will determine the extent to which the dark matter field fluctuates, and the void statistics may be sensitive to these quantities. Relating the topology of the point distribution to that of the smoothed density field is also an ongoing point of interest. Persistence provides a way of divining the significance of features found using PH. Alternatively, the smoothing scale used to convert a point distribution into a continuous field washes out small-scale holes and provides a measure of significance based on a physical scale. In addition, smoothing with a Gaussian kernel will sphericalize voids that are roughly equal in size to the smoothing volume, an effect that will not be present in the voids inferred from the point distribution. The fluid and particle descriptions will only match on large scales for objects significantly above the smoothing scale used to define the fluid. Relating the properties of voids obtained using the fluid and particle descriptions of matter would provide a link between the two different interpretations of the density field that generates spacetime curvature.

The topology of cosmological fields, the late-time galaxy distribution, cosmic microwave background, and weak lensing maps contain information beyond summary statistics that are commonly used by the community. Extracting this information, and comparing the results to mock data, provides an important consistency check of the ΛCDM model. Going further and inferring the ensemble average of topological summary statistics of random fields remains a long-standing goal.

## Figures and Tables

**Figure 7.**
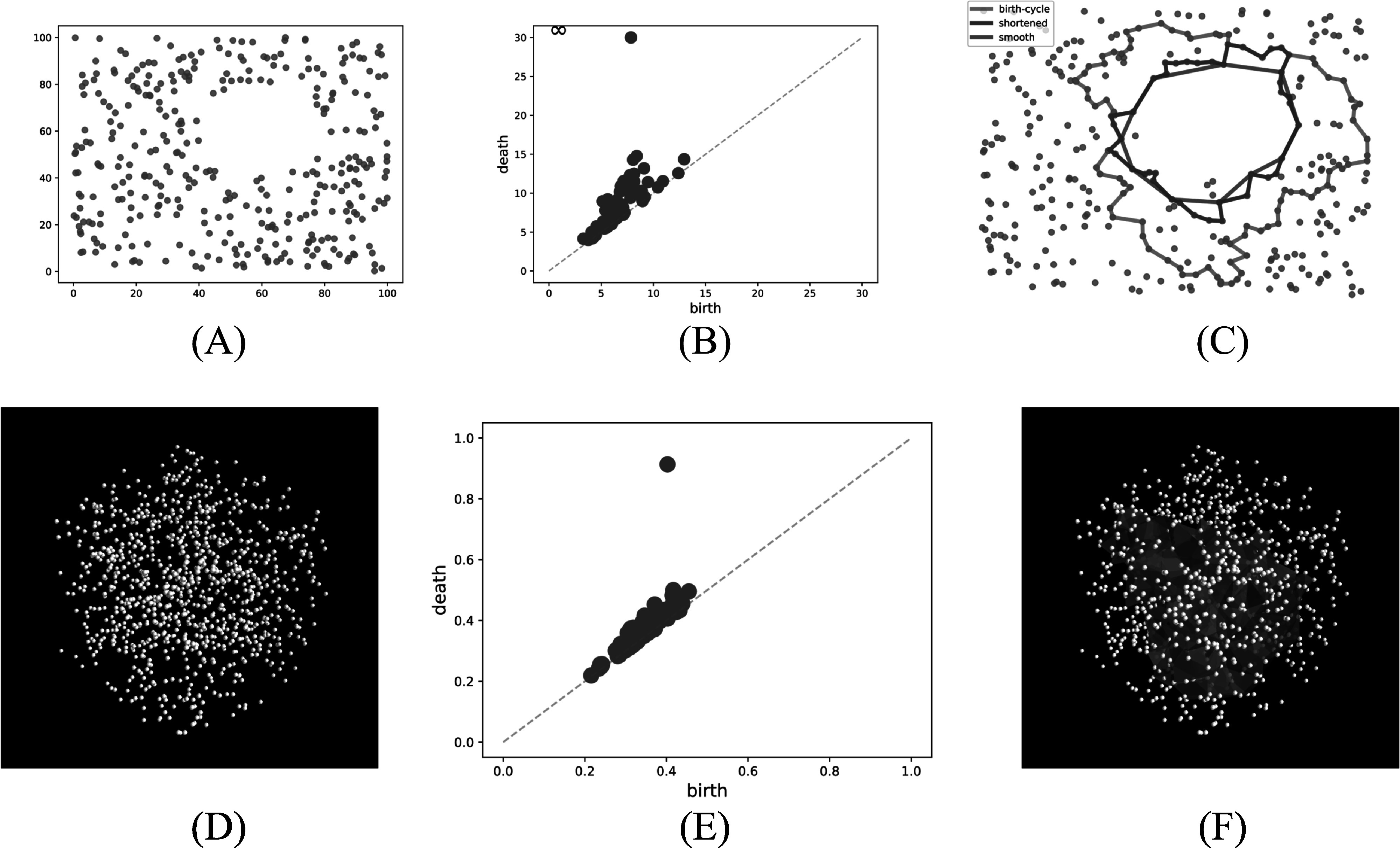
(A) A noisy discrete data set with one significant hole. (B) The births and deaths of holes are plotted as a persistence diagram. This example shows that only one hole has relatively high persistence. (C) Representative boundaries tighten as they are shortened by our algorithm. (D) A point cloud in three dimensions. No significant hole is visible. (E) PD shows that one feature has relatively high persistence. (F) Our algorithms improve geometric precision. The blue boundary is the birth cycle from the recursive algorithm. The red boundary is the smooth cycle after applying the greedy shortening and smoothing algorithms.

**Figure 8.**
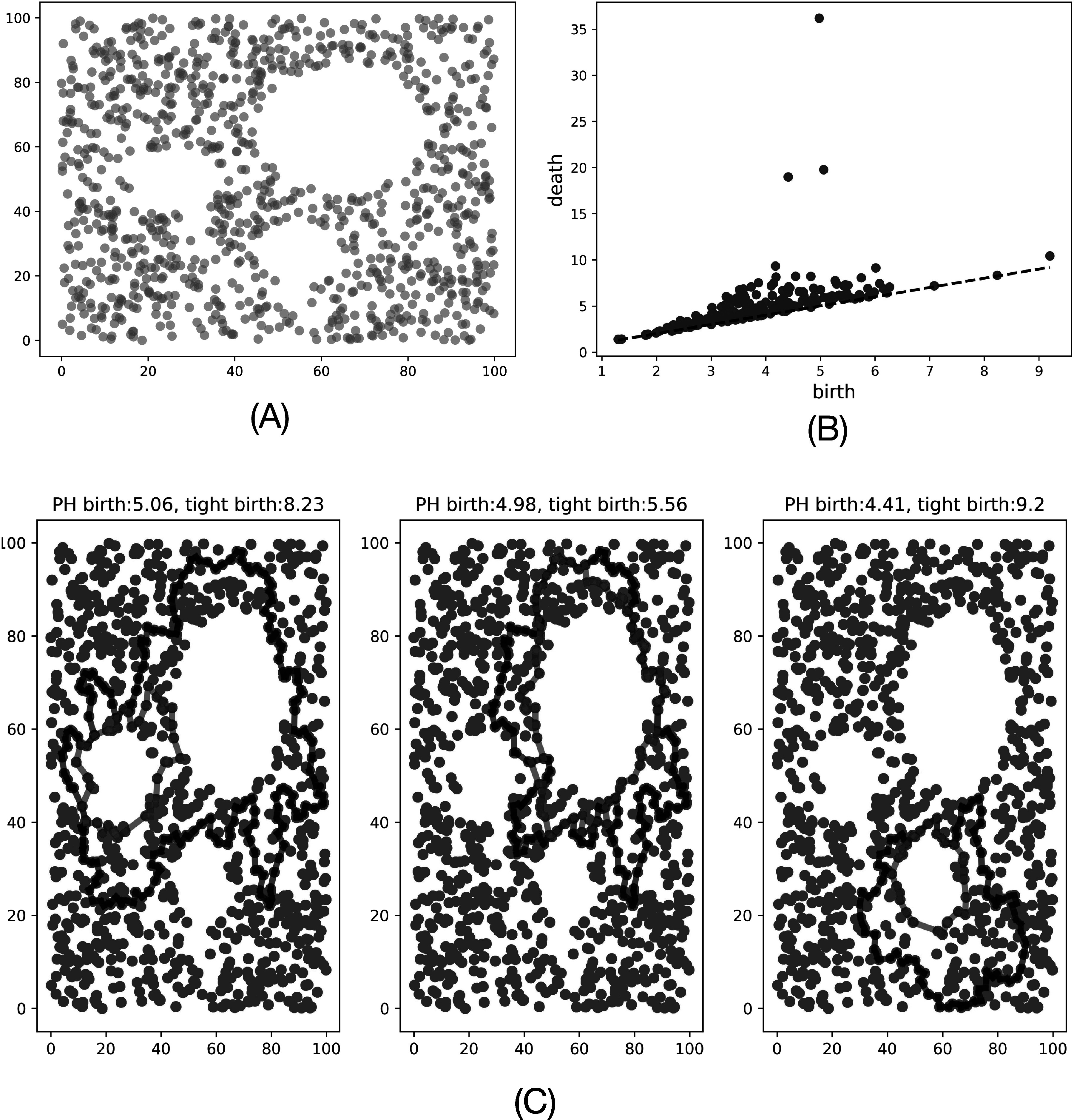
The number of tight representatives can be smaller than the number of significant features. (A) A simulated data set embedded in ${{\mathbb{R}}}^{2}$ with three distinct holes. (B) H_1_ PD shows there are three features with relatively high persistence. (C) Representatives from the matrix reduction algorithm are shown in black. Their birth parameters are 5.06, 4.98, and 4.41. Tight representatives after greedy shortening (*τ*_*u*_ = 10) are shown in red. Their birth parameters are 8.23, 5.56, and 9.2. Consequently, they have lower persistence, and some may be classified as nonsignificant depending on the persistence threshold.

**Figure 9.**
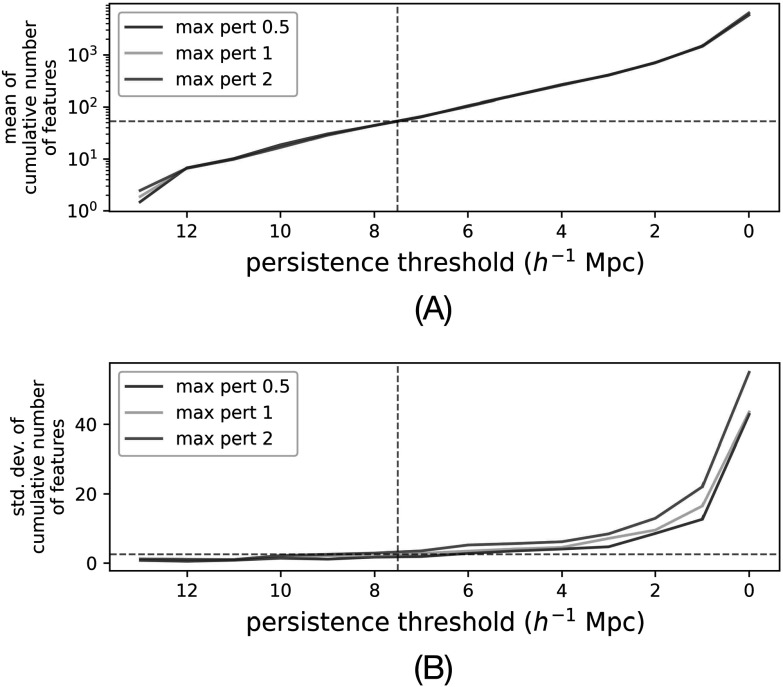
Number of H_2_ features is computed for 50 samples of three different perturbations of the data sets. Different colors show different magnitudes of the perturbation. (A) The mean number of features with persistence at most *x* decreases exponentially as *x* increases. (B) The standard deviation in the number of features is small and consistent across the three perturbations for persistence thresholds greater than 7.5 *h* ^−1^ Mpc (dashed vertical line).

**Figure 10.**
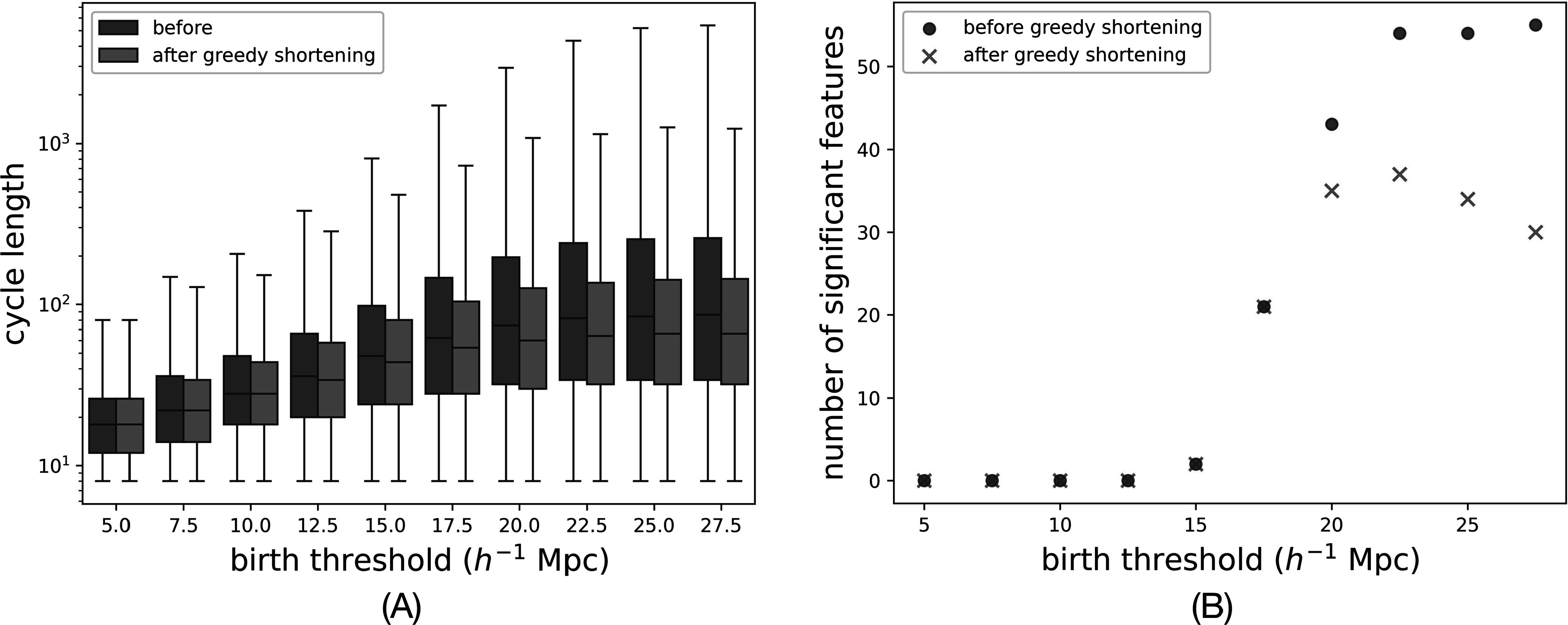
(A) Distributions of cycle lengths before and after greedy shortening at different birth thresholds (*τ*_*u*_). The distributions are similar for *τ*_*u*_ > 17.5 *h* ^−1^ Mpc. (B) The number of significant features around which we can find a representative after greedy shortening attains a maximum at *τ*_*u*_ = 22.5 *h* ^−1^ Mpc.

**Figure 11.**
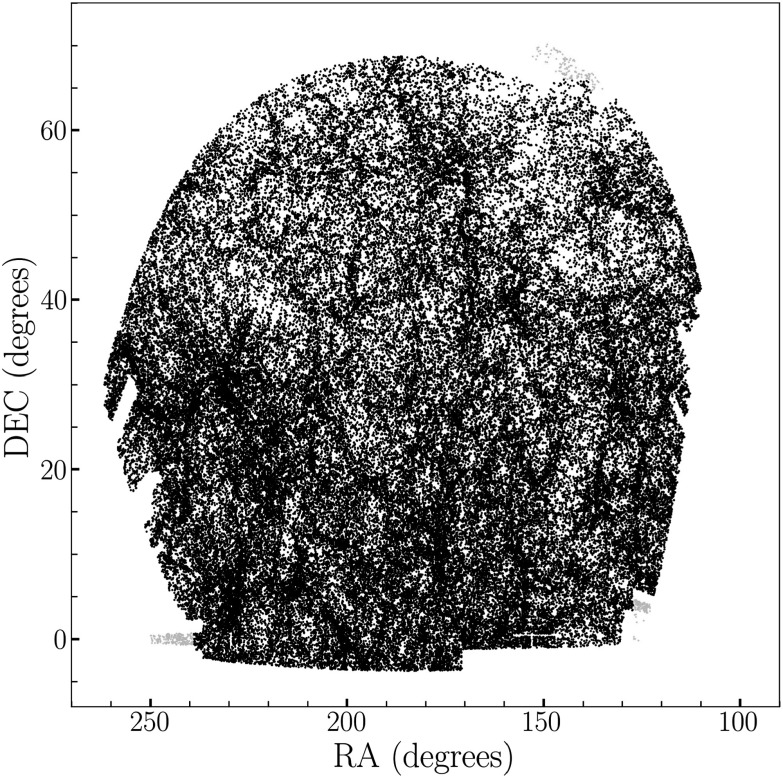
The angular distribution of SDSS galaxies on the sky. In the Appendix, we cut the gold points from the sample and reanalyze the data to test the sensitivity of the void properties to the boundary.

**Figure 12.**
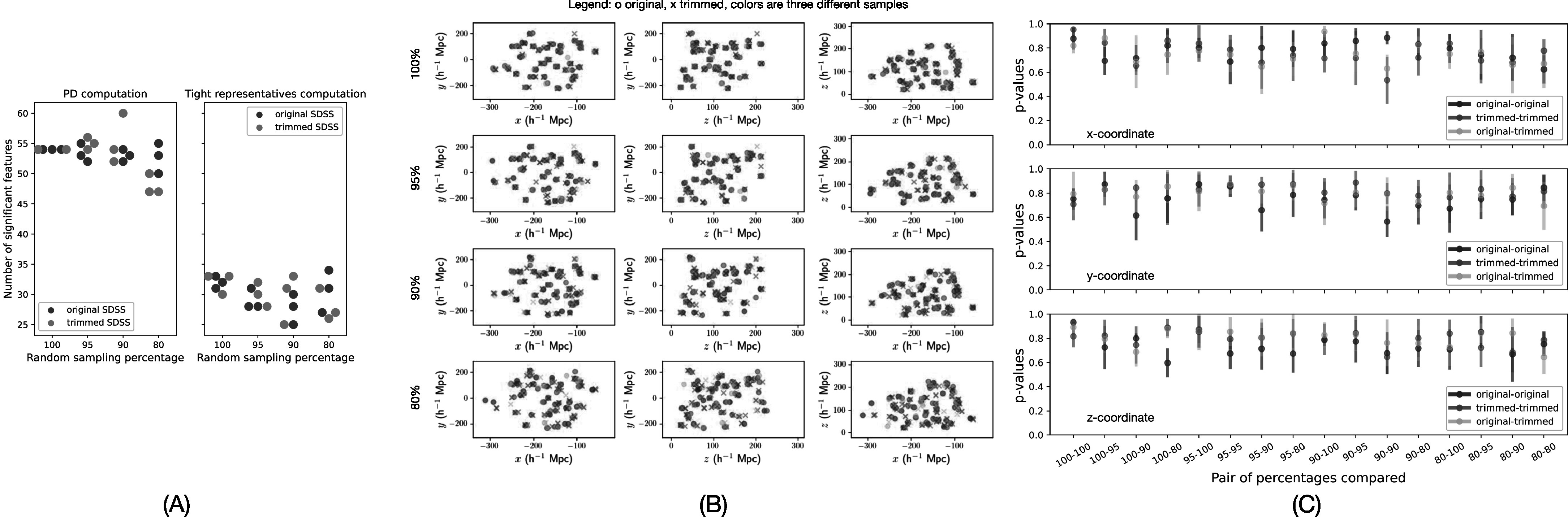
Comparing PH and tight representatives of different samples of original and trimmed data sets at different sampling percentages. (A) The number of significant features in PD and number of tight representatives computed when birth and persistence thresholds were kept the same across all samples (*τ*_*u*_ = 22.5 *h* ^−1^ Mpc, *ϵ* = 7.5 *h* ^−1^ Mpc). (B) Centroids of computed voids. (C) Results of the Mann–Whitney U rank test for comparing distributions of *x*, *y*, and *z* coordinates of void centroids for all pairwise combinations show that these distributions cannot be statistically distinguished (*p*-values ≫ 0.05).

**Figure 13.**
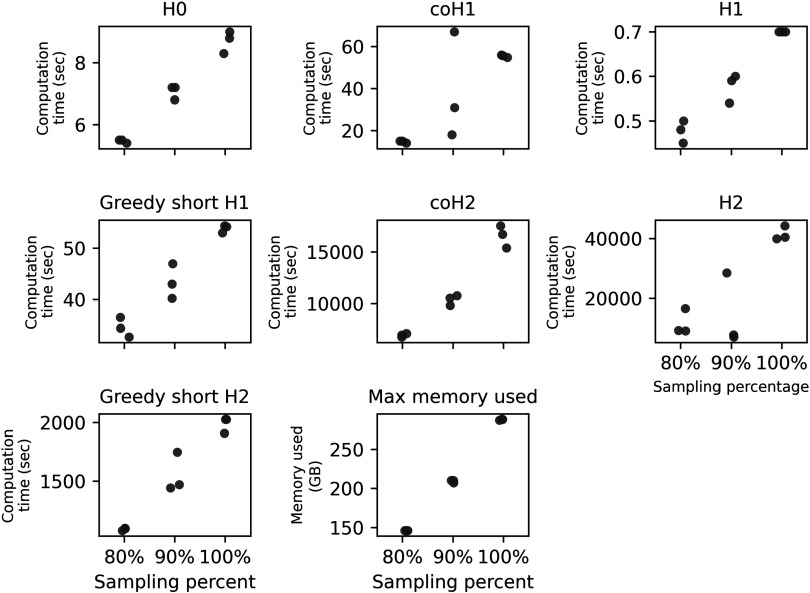
Computation times and memory for different under samples of the SDSS data set.

## Appendix A Persistent Homology Background and Terminology

Figure 7(A) shows an example of a discrete set of points or a point cloud. It has many gaps and holes, but one stands out as a larger hole compared to others. Dimension 1 PH computes the birth and death of holes across different spatial scales. This information is plotted as a PD. Figure 7(B) shows dimension 1 PD computed for the example in Figure 7(A). The persistence of a hole is defined as the difference between its death and birth. Those with higher persistence are more robust to noise in the data set. There is one feature (marked in red) with relatively higher dimension 1 persistence, as expected. Figure 7(C) shows multiple possible representative boundaries computed around the feature with maximum persistence.

## Appendix B PH Computation

Locations of topological voids were determined by computing tight representative boundaries of significant H_2_ features in the VR-filtration of the raw data. Features born at a spatial scale at most *τ*_*u*_ and with persistence, at least *p* were defined as significant. These parameters are user defined. PH was computed up to a threshold of *τ*_*u*_ + *p* using Dory (M. Aggarwal & V. Periwal 2024), which implements the matrix reduction algorithm to compute PH (A. Zomorodian & G. Carlsson 2005). Matrices in Dory are represented using compressed sparse row format, and the number of nonzero entries that can be represented is the upper limit of unsigned 32-bit int, *l* = 4294967295. However, computing PH for the galaxy data sets in this work resulted in matrices with more than *l* nonzero entries. Additionally, the number of nonzero columns also exceeds *l* in these computations. Both these issues were resolved by using a new sparse matrix format that increased the limits on the numbers of nonzero elements in a matrix and nonzero columns to *l* ^2^.

## Appendix C Computing Tight Representative Boundaries

Representative boundaries for all topological features were computed and shortened using algorithms introduced in M. Aggarwal et al. (2023). Briefly, columns of the matrix of reduction operations are used as the initial set of representatives. Representatives with birth parameters less than the user-defined birth threshold are greedily shortened by summing (modulus 2) boundaries pairwise that result in a maximal reduction in lengths.

We now explain why the number of computed tight representatives can be less than the number of significant features. Figure 8(A) shows a simulated data set in two dimensions with three holes that visually stand out. The H_1_ PD in Figure 8(B) shows that there are three features with relatively high persistence. Figure 8(C) shows representative boundaries computed by the matrix reduction algorithm (black curves) around these features. The birth parameters of these features are 5.06, 4.98, and 4.41. Geometrically, the birth parameter of a feature implies that the algorithm finds a cycle around the feature with the length of its longest edge equal to the birth parameter. Here we compute tight representatives with birth threshold *τ*_*u*_ = 10. Representatives with birth parameters less than 10 are summed modulus 2 for greedy shortening. This process results in new representatives that may have the same or larger birth parameters but smaller than *τ*_*u*_ = 10. Figure 8(C) shows the representatives after greedy shortening in red. They are geometrically tighter around the features; however, their birth parameters are 8.23, 5.56, and 9.2, all larger than the birth parameters of these features from the matrix reduction algorithm (5.06, 4.98, and 4.41, respectively). The death parameter remains unchanged. As a result, the death–birth of tight representatives might be less than the death–birth of the corresponding features that are computed by the matrix reduction algorithm. Consequently, the persistence of some of the tight representatives around significant features may be below the persistence threshold, classifying them as nonsignificant. The same reasoning follows for features of higher dimensions.

After greedy shortening, tight representatives that can potentially contain topologically significant features are stochastically shortened as follows. A local cover of the representative boundary is computed as the smallest hyperrectangle with points of the boundary inside or on it. Stochasticity in the cover is introduced by perturbations and permutations as follows. First, *n*_pert_ perturbations (a user-defined parameter) are constructed. The maximum perturbation parameter is initialized as Δ_*m*_ = *p*/3. For each point ${p}_{i}$, we denote the distance of its nearest neighbor by *d*_*i*_. Each point ${p}_{i}$ is perturbed randomly in a neighborhood of radius ${r}_{i}=\min \{{d}_{i}/3,{{\mathrm{\Delta }}}_{m}\}.$ *n*_pert_ perturbations are constructed, and PH up to *τ* = *τ*_*u*_ + *p* is computed for each perturbation. If the number of significant features in each of the *n*_pert_ perturbations is not the same as the number of significant features in the unperturbed cover, we halve Δ_*m*_ and repeat. Otherwise, we are done with constructing perturbations of the cover. For each perturbation of a cover, we create *n*_perm_ permutations (user-defined parameter) as follows. A list of edges of lengths up to *τ* is constructed that is sorted by their lengths. The lengths are rounded to decimal precision *r* (user-defined parameter). The order of the sorted edges defines the order of columns in the boundary and coboundary matrices for PH and representative boundary computation. Permutations are constructed by permuting the order of edges of the same length. Note that the number of possible unique permutations might be less than *n*_perm_. PH is computed for (at most) *n*_pert_ × *n*_perm_ sorted lists of edges, for each cover, up to *τ*. Representative boundaries born at a spatial scale at most *τ*_*u*_ are also computed. From all computations for a cover, a set of all boundaries that possibly wrap around significant feature(s) is determined. Minimal boundaries are selected from these sets, and they are smoothed using an algorithm detailed in the S1 text of M. Aggarwal et al. (2023).

## Appendix D Choosing Parameters to Define Significant Features

For all data sets in this work, we defined significant features as those born before *τ*_*u*_ = 22.5 *h* ^−1^ Mpc and with persistence at least *p* = 7.5 *h* ^−1^ Mpc. Here, we explain these choices.

### D.1. Persistence Threshold

First, we decide the value of the persistence threshold. A higher persistence implies higher robustness to noise or perturbations in the data set. Hence, we perturbed the location of each galaxy randomly in a ball of radius Δ and computed PH. This was done for 50 samples for each of Δ = {0.5, 1, 2} *h* ^−1^ Mpc. Then, we count the number of H_2_ features. Figure 9(A) shows the mean of cumulative counts of features for the different values of Δ. For instance, a point (*x*_*_, *y*_*_) on this curve informs that there are *y*_*_ nontrivial H_2_ features (averaged over the 50 samples) with persistence at most *x*_*_. In other words, the *x*-axis is the persistence threshold. As expected, the number of features increases as the persistence threshold decreases (notice that the *x*-axis is reversed in the plot). Figure 9(B) shows the standard deviation in the cumulative number of features. In concordance with the implication of higher persistence, the standard deviation is lower at a higher persistence threshold. Moreover, at low persistence thresholds, standard deviations are inconsistent across the different values of Δ. At *x* = 7.5, the standard deviation is small and consistent across the different perturbations. At higher thresholds, the number of features decreases exponentially (see Figure 9(A)). Hence, we choose *p* = 7.5 *h* ^−1^ Mpc as a conservative choice for the persistence threshold.

### D.2. Birth Threshold

Given a persistence threshold, we now decide the birth threshold. Figure 10(A) shows the distribution of lengths of representatives, before and after greedy shortening, for different values of birth thresholds. We note that for birth thresholds greater than 22.5 *h* ^−1^ Mpc, the distributions of cycle lengths after greedy shortening are similar. Further, we compute the number of significant features at each birth threshold. Figure 10(B) shows that the peak in the number of significant features that are enclosed within a shortened representative is at 22.5 *h* ^−1^ Mpc. Hence, we choose *τ*_*u*_ = 22.5 *h* ^−1^ Mpc as the birth threshold to define significant features.

## Appendix E Sensitivity of Void Properties to Data Variations

In this section we address two issues. First, we trim a small number of protruding surfaces in the angular footprint of the SDSS. Topological statistics are often particularly sensitive to complicated data boundaries, because the underlying point distribution is inhomogeneously sampled in these regions. Or in the language of smoothed fields, the underlying density field is subject to a larger noise component in the absence of data that is cut by the survey boundaries. We test that the voids found in this work are not significantly compromised by the boundary, by generating a slightly simpler angular footprint. In Figure 11 we present the full data used in the main body of the paper, but the gold points are cut in this section. In what follows, we call the new data set “trimmed” (see black points, Figure 11) and the nontrimmed “original”. These particular regions were chosen to maximize the volume to the surface area of the data and make the footprint closer to a spherical cap on the sky. If the data is bounded by a complicated mask geometry, then spurious triangulated meshes can be generated that cross the masked regions.

Second, we analyze the robustness of the voids found by undersampling both original and trimmed data sets. We compute and compare PH and tight representatives of original and trimmed data sets that are undersampled at three different sampling percentages of *p* = 95%, 90%, and 80% (three samples each). We call original and trimmed data sets as catalogs. Then, the comparison of catalog1-catalog2 at percentages *p*_1_–*p*_2_ is the Mann–Whitney U rank test of distributions of centroids of a sample of catalog1 at percentage *p*_1_ with that of a sample of catalog2 at percentage *p*_2_. This is done for all pairwise combinations of samples (six combinations if catalog1 = catalog2, and nine combinations otherwise), resulting in a distribution of *p*-values of statistical significance.

We also compare computations for three samples of full data sets (100%) such that the order of edges of the same lengths in the matrix reduction algorithm is shuffled. Such a shuffling will have no effect on the PD, but the computed tight representatives might be different. These samples are shown as 100% shuffling in the figures. The birth and persistence thresholds for all data sets are *τ*_*u*_ = 22.5 *h* ^−1^ Mpc and *ϵ* = 7.5 *h* ^−1^ Mpc, respectively.

Figure 12(A) shows the number of significant features from PD computation and the number of computed tight representatives. The number of significant features from PD computation in the full original and trimmed data sets are exactly the same, as expected. The number of computed tight representatives varies between 30 and 33 for the different shuffled full data sets of original and trimmed. The variance in the number of significant features and the number of tight representatives increases at percentages 90 and 80.

We next compare the centroids of the computed tight representatives. Figure 12(B) shows the coordinates of the centroids for all cases—the original is shown by the o marker, trimmed by the x marker, and different colors show the different samples. We note that the majority of o and x match, and the pattern is also similar as the sampling percentage decreases (comparing top to bottom). We quantify this by conducting the Mann–Whitney U rank test for *x*, *y*, and *z* coordinates. Figure 12(C) shows that the *p*-values from this significance test are ≫0.05, indicating that these distributions cannot be statistically distinguished.

## Appendix F Information for Void Centroids

Table 1 shows centroids, *R*_eff_, and sphericity of the tight representatives computed for PH voids in SDSS. The three different samples (Sample 0, 1, and 2) correspond to tight representatives computed for three different bijective mappings of galaxies to integers. Such permutations will not affect the PDs but can result in differences in the tight representative computation due to the stochasticity of our algorithm. Nevertheless, Figure 12(C) shows that these differences are not significant—*p*-values for 100–100 comparison for both original and trimmed are much greater than 0.05 and very close to 1. The main text uses the results of Sample 1.

**Table 1:** SDSS Void Centroids of the Computed Tight Representatives

Sample 0					Sample 1					Sample 2				
R.A.	Decl.	*z*	*R*_eff_	*ψ*	R.A.	Decl.	*z*	*R*_eff_	*ψ*	R.A.	Decl.	*z*	*R*_eff_	*ψ*
236.62	26.3	0.101	43.62	0.78	235.8	25.63	0.1013	41.63	0.78	236.44	26.35	0.1008	44.04	0.74
172.34	33.83	0.0941	51.86	0.75	172.31	34.23	0.0941	54.84	0.73	174.59	34.82	0.0938	46.64	0.77
125.57	44.47	0.0872	44.05	0.8	211.55	43.61	0.0539	47.69	0.77	125.64	46.12	0.0885	42.87	0.8
196.07	21.55	0.0975	48.33	0.75	125.14	44.77	0.0879	41.86	0.78	155.34	21.14	0.0926	43.15	0.77
224.22	4.4	0.1048	33.8	0.8	217.43	9.46	0.0752	40.69	0.76	213.5	43.31	0.0522	46.3	0.74
210.25	43.62	0.0519	45.78	0.77	223.64	3.97	0.1051	34.89	0.78	217.26	9.45	0.0757	40.77	0.74
216.85	10.13	0.0754	39.14	0.74	193.08	22.35	0.0977	43.0	0.8	224.47	4.25	0.1035	33.52	0.84
154.99	22.23	0.0965	43.53	0.75	213.6	30.73	0.0953	40.3	0.81	193.14	21.55	0.0975	44.04	0.81
134.56	46.17	0.0622	36.01	0.8	156.97	21.87	0.0947	40.82	0.78	213.39	30.56	0.0972	41.9	0.8
212.36	29.64	0.0981	41.9	0.82	201.06	20.25	0.0565	34.75	0.8	134.12	45.49	0.062	35.97	0.8
149.28	12.51	0.0911	39.62	0.78	133.31	45.48	0.0608	35.39	0.79	157.98	19.34	0.0732	32.58	0.81
157.14	29.53	0.1061	33.64	0.85	148.39	13.01	0.0916	38.77	0.82	157.26	28.93	0.1057	33.84	0.83
199.41	19.34	0.0544	35.75	0.8	239.96	12.7	0.0823	34.68	0.83	199.66	19.4	0.0555	34.17	0.79
157.37	18.15	0.0732	35.9	0.81	142.68	17.49	0.0618	35.19	0.83	143.46	17.33	0.0607	33.89	0.82
155.57	30.59	0.0604	38.61	0.79	157.08	29.31	0.1058	33.3	0.86	233.75	13.08	0.0652	34.23	0.83
142.42	17.74	0.062	35.29	0.79	155.28	33.37	0.0628	38.15	0.77	179.27	28.27	0.0644	27.86	0.81
152.01	38.67	0.0992	28.13	0.86	152.84	38.95	0.0996	29.47	0.79	152.83	30.89	0.0607	36.6	0.81
126.78	42.1	0.0728	29.74	0.83	179.29	29.21	0.0634	27.57	0.84	169.64	38.13	0.0532	30.92	0.83
234.06	13.46	0.067	29.46	0.86	126.73	41.27	0.0725	30.39	0.83	152.83	38.78	0.0972	27.37	0.86
177.87	28.67	0.0642	27.26	0.83	233.83	10.2	0.0641	27.92	0.85	135.28	9.31	0.0999	30.76	0.8
194.05	14.33	0.1058	32.79	0.82	232.06	44.66	0.0828	31.85	0.81	195.01	60.23	0.0844	33.1	0.88
192.74	60.28	0.0826	33.17	0.86	192.45	60.6	0.0847	34.79	0.84	214.6	12.66	0.0501	30.53	0.85
189.11	35.92	0.0751	31.07	0.82	193.04	14.69	0.1061	33.78	0.82	193.07	14.78	0.1062	35.24	0.82
217.38	10.37	0.0466	30.04	0.83	211.92	11.65	0.052	28.05	0.85	231.51	44.58	0.0837	31.65	0.86
201.79	27.99	0.0811	26.85	0.84	134.48	10.15	0.0996	31.12	0.79	126.12	41.95	0.0735	28.08	0.87
230.87	45.3	0.0838	30.41	0.88	200.33	28.02	0.0822	27.7	0.87	189.06	37.16	0.0773	28.06	0.85
136.2	8.66	0.1004	28.79	0.82	227.21	25.87	0.0936	30.07	0.78	201.51	28.02	0.0812	26.9	0.81
132.45	31.73	0.0345	27.31	0.89	189.94	37.18	0.0779	26.84	0.87	227.76	24.92	0.096	28.03	0.81
151.46	36.82	0.0858	25.5	0.91	151.04	38.06	0.0841	27.28	0.89	150.8	37.11	0.0852	26.49	0.88
141.57	47.16	0.0992	18.82	0.94	132.26	30.77	0.0341	27.66	0.88	127.11	27.94	0.0613	26.51	0.81
205.06	24.72	0.1	22.88	0.9	206.11	25.47	0.0987	22.12	0.91	133.42	32.51	0.0353	26.87	0.92
⋯	⋯	⋯	⋯	⋯	142.38	46.01	0.0993	19.68	0.92	205.26	24.53	0.0991	25.44	0.91
⋯	⋯	⋯	⋯	⋯	⋯	⋯	⋯	⋯	⋯	141.95	45.46	0.0995	20.05	0.91

## Appendix G Supplementary Information

### G.1. Computation Cost

Figure 13 shows computation costs for the different steps of computing tight representatives (up to a spatial scale of 30 *h* ^−1^ Mpc and greedy shortening).
